# No effects of caffeine on cycling to exhaustion and perceptual responses in non-caffeine-restricted subjects

**DOI:** 10.1080/15502783.2025.2534131

**Published:** 2025-07-24

**Authors:** Matthias Weippert, Martin Behrens, Martin Schlegel, Tom Schröder, Moritz Tillmann, Nelly Rühe, Robert Römer, Anett Mau-Möller, Sven Bruhn

**Affiliations:** a University of Rostock, Institute of Sport Science, Rostock, Germany; b University of Applied Sciences for Sport and Management Potsdam, Potsdam, Germany; c University Medical Centre, Department of Orthopedics, Rostock, Germany

**Keywords:** withdrawal reversal hypothesis, effort, time to exhaustion, endurance performance, fatigue, time perception

## Abstract

**Introduction:**

Caffeine has been shown to improve endurance performance probably primary due to its pharmacological effects in the central nervous system modifying, among others, the perceptual responses during exercise. However, most studies proving the performance-enhancing effects of caffeine utilized an experimental caffeine restriction phase prior to the measurement sessions. Therefore, the effects of 2.5 and 6 mg*kg^−1^ oral caffeine ingestion on endurance performance, perceptual, affective, and cognitive responses during exercise, as well as time perception, were investigated in participants following their normal “ad libitum” daily diet.

**Methods:**

Two double-blinded, randomized placebo-controlled cross-over studies were performed to test the effect of 2.5 (*N* = 35, age: 23.3 ± 3.5 years, habitual caffeine consumption of 106 ± 89 mg*day^−1^) and 6.0 mg*kg^−1^ (*N* = 21, age: 21.2 ± 2.3 years, habitual caffeine consumption of 87 ± 64 mg*day^−1^) oral caffeine ingestion on time to exhaustion (TTE), perceived fatigue, perceptual-discriminatory (effort perception, physical strain), affective-motivational (affective valence, arousal, dominance, motivation, boredom), and cognitive-evaluative responses (decisional conflict, attentional focus) as well as time perception (time production and estimation) and heart rate during cycling at 65% peak power. Participants were low-to-moderate caffeine consumers (one participant in each study reported no habitual caffeine intake) and asked to follow their regular “ad libitum” diet without any restrictions regarding caffeinated beverages and/or food during the studies.

**Results:**

Neither a dose of 2.5 nor of 6.0 mg*kg^−1^ was found to be superior to placebo with respect to TTE, perceived fatigue, the perceptual-discriminatory, affective-motivational, and cognitive-evaluative responses to exercise, as well as time perception.

**Conclusion:**

Both dosages of caffeine had no effect on TTE, perceived fatigue, perceptual-discriminatory, affective-motivational, and cognitive-evaluative responses to exercise, as well as on time perception and heart rate in low-to-moderate caffeine consumers without a prior experimental caffeine restriction phase. The findings suggest that caffeine´s positive effects on endurance performance and perceptual responses to exercise found in previous studies might be partly explained by the reversal of adverse effects induced by a prior caffeine restriction phase.

## 1. Introduction

Oral caffeine ingestion has been shown to acutely increase endurance performance probably primary through its action on the central nervous system [1]. Due to its function as an adenosine receptor antagonist, caffeine might modulate different exercise-related perceptions like fatigue, effort, and exercise-induced pain, as well as affective valence and arousal [2–4]. However, the effects of caffeine on performance and perceptual responses have been almost exclusively investigated with study designs using a caffeine restriction phase prior to the experiments. A recent umbrella review confirmed a lack of knowledge regarding the influence of caffeine habituation and the minimal ergogenic dosage [5]. Others also noted that the evidence base exploring both caffeine habituation and withdrawal strategies in athletes is surprisingly small [6] or support the notion of inconsistencies regarding the ergogenic effects with respect to habituation [7], while the latest review questions a detrimental effect of habituation on caffeine´s ergogenicity [8]. Only few studies have addressed the effects of caffeine habituation from a truly experimental perspective [9] or employed an ecological valid design without a prior caffeine restriction phase [10,11]. For instance, Irwin et al. [10] compared the acute effects of caffeine on cycling performance after a 4-day withdrawal and a 4-day non-withdrawal period in participants with a relatively high habitual caffeine consumption (240 ± 162 mg*day^−1^). While the authors detected no difference for the performance-enhancing effect of an acute caffeine ingestion after the two phases, a rebound effect cannot be ruled out, since testing followed an overnight phase of caffeine restriction (≥12 h). In the small sample of athletes investigated by Van Soeren and Graham [11], it is striking that in the non-restricted condition, the plasma concentration of caffeine before the experimental test was similarly low or even lower than in the restriction condition. This also suggests a kind of withdrawal before the testing and a rebound effect due to acute caffeine administration, especially when considering the reported high habitual caffeine intake of the participants (761 ± 12 mg*day^−1^). Thus, it remains unclear whether the empirical findings of the performance-enhancing effects of caffeine might be in part related to an artificially or coincidentally induced withdrawal symptomatology prior to the experiments, as it has been suggested for studies dealing with the effects of caffeine on cognitive performance [12], and whether caffeine has also positive effects on endurance performance and perceptual responses to exercise under more ecologically valid conditions without a caffeine restriction phase. Further, the extrapolation of literature findings to females should be done with caution due to the limited number of studies including a sufficient number of female participants [8,13].

Furthermore, the effect of caffeine on the dynamics of perceived fatigue as well as perceptual-discriminatory, affective-motivational, and cognitive-evaluative responses to exercise [4,14], as well as on time perception during endurance exercise, has not been studied to a full extent so far. This is surprising, given that the performance-enhancing effects of caffeine have been attributed to its action on perceptual responses to exercise, e.g. a reduction in the effort perception during exercise [2,15].

While most studies showing positive effects on endurance performance using dosages of 3–6 mg*kg^−1^ body mass, the minimal effective dose may be as low as 2 mg*kg^−1^[1]. These lower doses correspond to a typical dietary intake of caffeine, e.g. two cups of coffee (500 ml) contain around 120–180 mg caffeine [16,17], resulting in 1.7–2.6 mg*kg^−1^ for a 70 kg person.

Therefore, two studies were performed investigating the effects of (i) 2.5 mg*kg^−1^ and (ii) 6 mg*kg^−1^ body mass oral caffeine ingestion on time to exhaustion (TTE), perceived fatigue, perceptual-discriminatory (effort perception, physical strain), affective-motivational (affective valence, arousal, dominance, motivation, boredom), and cognitive-evaluative responses (decisional conflict, attentional focus) as well as time perception (time production and estimation) and heart rate (HR) during cycling at 65% peak power without a prior caffeine restriction phase. It was hypothesized that caffeine intake increases TTE and positively influences the perceptual-discriminatory, affective-motivational, and cognitive responses to exercise as well as time perception during constant–load cycling until exhaustion in physically active subjects.

## 2. Methods

Both studies were performed in compliance with the Declaration of Helsinki, and approval of the local ethics committee at the University of Rostock was obtained (registration number: A 2020–0067). All participants gave their written informed consent to take part.

### 2.1. Participants

To the best of our knowledge, there is only one study investigating the effect of oral caffeine ingestion (5 mg*kg^−1^) on ratings of perceived exertion (RPE) during cycling to exhaustion at 80% of maximal oxygen uptake (VO_2_max) with a prior caffeine withdrawal period ≤12 h and specific time intervals after caffeine ingestion (1, 3, 6 h before cycling) in a sample of caffeine users and non-users [18]. We used the reported RPE values (15 min after the start, ingestion interval before cycling 1 h) of both groups to calculate the average effect of caffeine on RPE. Pooled data gave a large effect size (*Cohen´s f = 0.4*) for the effect of caffeine on RPE during exercise. Due to the smaller dosage, a medium effect size (*Cohen`s f = 0.3*) with regard to the same outcome was expected for study 1 (2.5 mg*kg^−1^ caffeine) and a similar, large effect size (*Cohen´s f = 0.4*) for study 2 (6 mg*kg^−1^ caffeine). Consequently, the a priori sample size calculations (α level of 0.05 and a power (1−β) of 0.95, F-test family, repeated measures analysis of variance (RM-ANOVA)), within factors) with G.Power 3.1.9.7 (University of Kiel) revealed that 32 subjects would be required for study 1 and 18 subjects for study 2. Exclusion criteria for both studies were a history of musculoskeletal, neurological or cardiovascular disorders or any other chronic or acute conditions that preclude participation in the experiment. Before the first visit to the laboratory, the Physical Activity Readiness Questionnaire (PAR-Q) was used to check for potential medical reasons for exclusion. The habitual caffeine consumption was assessed and calculated with a caffeine consumption questionnaire developed for adolescents and young adults [16]. Participants were low to moderate caffeine consumers (Tables 1 and 2) and asked to follow their normal diet during the studies.

**Table 1.** Participant characteristics (means ± standards deviation) of study 1 (2.5 mg*kg^−1^ caffeine).

Sex	N	Body height [cm]	Body weight [kg]	Self-reported caffeine consumption [mg*day^−1^]	Age [years]	BMI [kg*m^−2^]	PPO [W]	HR_peak_ [min^−1^]
Females	17	171 ± 5	65 ± 9	106 ± 96	22.2 ± 2.0	22.1 ± 2.5	268 ± 37	191 ± 11
Males	18	181 ± 5	76 ± 9	105 ± 84	24.3 ± 4.9	23.1 ± 2.4	354 ± 50	199 ± 7

BMI: Body mass index, PPO: Peak power output, HR_peak_: Maximal heart rate during the incremental test.

**Table 2.** Participant characteristics (means ± standard deviations) of study 2 (6 mg*kg^−1^ caffeine).

Sex	N	Body height [cm]	Body weight [kg]	Self-reported caffeine consumption [mg*day^−1^]	Age [years]	BMI [kg*m^−2^]	PPO [W]	HR_peak_ [min^−1^]
Females	11	168 ± 3	59 ± 5	85 ± 68	21.5 ± 2.5	20.9 ± 1.8	249 ± 28	191 ± 9
Males	10	184 ± 7	78 ± 6	88 ± 63	20.8 ± 2.0	23.0 ± 1.5	348 ± 37	190 ± 11

BMI: Body mass index, PPO: Peak power output, HR_peak_: Maximal heart rate during the incremental test.

#### 2.1.1. Study 1 (2.5 mg*kg^−1^)

To increase statistical power and consider potential dropouts, 41 recreationally active subjects (age: 23.2 ± 3.9 years, body height: 175.6 ± 7.9 cm, body weight: 69.4 ± 11.4 kg, 21 eumenorrheic females) were recruited. Participants were physically healthy and active in diverse individual and team sports. Activity level was assessed with the short version of the International Physical Activity Questionnaire (IPAQ) and gave an average number of 2.7 ± 1.9 vigorous activities per week (average activity duration: 59.6 ± 50.4 min) and 3.3 ± 1.6 moderate activities per week (average activity duration: 59.1 ± 93.2 min). Due to medical problems unrelated to our experiment, six subjects had to withdraw from the study before or after the first experimental visit. Thus, data of 35 participants (Table 1) with an average daily caffeine consumption of 106 ± 89 mg (range: 0–296 mg) were included in the analyses. One of these 35 participants reported that she did not habitually consume caffeine.

#### 2.1.2. Study 2 (6 mg*kg^−1^)

To increase statistical power and consider potential dropouts, 22 recreationally active subjects (age: 21.9 ± 4.2 years, body height: 176.1 ± 9.3 cm, body weight: 68.7 ± 10.8 kg, 11 eumenorrheic females) were recruited. Participants were physically healthy and active in diverse individual and team sports. Activity level was assessed with the short version of the IPAQ and gave an average number of 3.5 ± 1.8 vigorous activities per week (average activity duration: 85.0 ± 34.6 min) and 3.9 ± 1.9 moderate activities per week (average activity duration: 118.4 ± 197.5 min). Due to medical problems unrelated to our experiment, one subject had to withdraw from the study before the first experimental visit. Thus, data of 21 participants (Table 2) with an average daily caffeine consumption of 87 ± 64 mg (range: 0–245 mg) were included in the analyses. One out of these 21 participants stated that she did not habitually consume caffeine.

### 2.2. Study designs and procedures

**Figure 1.**
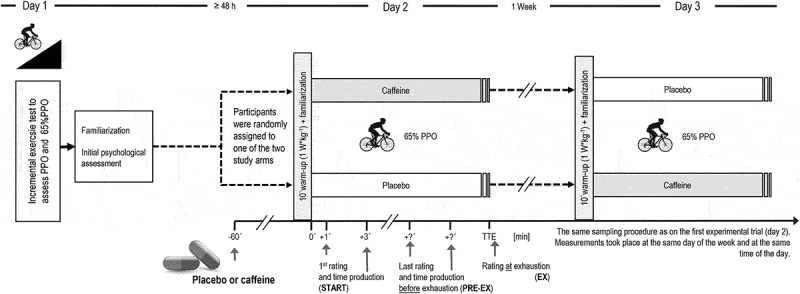
Experimental design of the two studies. PPO: Peak power output, TTE: Time to exhaustion.

Participants visited the laboratory on three occasions during both studies (familiarization session, two experimental trials, respectively). During the familiarization sessions, peak power output (PPO) was determined using an incremental cycling test (initial load: 1 min at 50 W increased by 25 W*min^−1^, preceded by a 5 min warm-up at 50 W) until volitional exhaustion at least 48 h before the first experimental session (Figure 1). Before and after the incremental cycling test, participants were thoroughly familiarized with the experimental procedures and rating scales using standardized written instructions. They were further instructed to refrain from strenuous exercise at least 48 h prior the experimental days and to maintain their physical activity pattern and diet throughout the study. A randomized placebo-controlled, double-blind crossover design was applied for both studies. Participants completed two constant-load cycling trials on a SRM ergometer (Schoberer Messtechnik, Jülich, Germany) at 65% PPO until exhaustion in constant laboratory conditions (ambient temperature: 18–21°C, humidity: 31–34%) in each study. During the experimental sessions, caffeine (CAF) or placebo (PLA) (white maize flour) was administered 60 min before the start in gelatin capsules (Detrade UG, Stuhr, Germany) and the conditions were randomly assigned using the www.randomizer.org website. Participants were informed that no verbal encouragement would be given by the investigators during the experimental sessions to minimize investigator-related bias. Female participants reported the day of their menstrual cycle on each experimental days via an online questionnaire to consider the menstrual cycle phase as a potential confounder within in the statistical analyses.

To elucidate the development of perceived fatigue as well as the perceptual-discriminatory, affective-motivational, and cognitive-evaluative responses to exercise [4,19,20], the respective rating scales were presented on a screen in front of the participants (see also the section *rating scales*).

Intra-participant testing was conducted at weekly intervals at the same time of the day to minimize circadian and activity variations. On each experimental day, subjects were repeatedly familiarized with the rating scales during the 10 min warm-up (1 W*kg^−1^).

Although it was assumed that participants were highly intrinsically motivated, a financial reward of 50 € for the best ranked cumulated performance, i.e. sum of the time achieved in the TTE trials in the two experimental conditions, was announced.

### 2.3. Performance and physiological data

TTE was measured with a manual stopwatch (Schütt GmbH, Germany). Each trial was stopped at volitional exhaustion or if participants were no longer able to hold a cadence ≥ 60 rpm for more than 10 s. HR was recorded during each exercise session using a RS 800 HR monitor (Polar Electro Oy, Finland). Absolute and relative HR (percentage of individual peak HR during the incremental exercise test) were used to assess objective physiological strain during the trials.

### 2.4. Rating scales

To elucidate the dynamics of perceived fatigue as well as the perceptual-discriminatory, affective-motivational, and cognitive-evaluative responses to exercise during the TTE trials, different rating scales were presented on a 46-inch flat screen (Samsung Inc. Model LE46B530P7WXZG) in front of the cycle ergometer (distance from the bars: 1.4 m, height: 1.1 m) at 5-min intervals using Microsoft® Power Point 2016. The rating interval and the cycling time were not disclosed to the participants. Ten different rating scales were applied (completion time: 1 min).

The dynamics of overall perceived fatigue was assessed via a category ratio (CR)-10 scale with “0” representing “not fatigued/exhausted at all” and “10” representing “very exhausted/fatigued” [21].

The perceptual-discriminatory dimension was addressed using two different 15-point scales (6–20). To assess perceived effort, participants were asked to evaluate how difficult the task is or how strenuous it is to maintain cycling (“How hard do you have to exert yourself to maintain the power output? How strenuous is it to move your legs and how difficult is it for you to breathe?”). They were explicitly informed that other sensations accompanying cycling (e.g. exercise-induced muscle pain, breathing discomfort) should not be taken into account in their evaluation of effort, to avoid confounding with aspects of perceived physical strain. The intensity of exercise-induced perceived physical strain was assessed with another 15-point scale. Here, participants were asked to rate, how strong the physical sensations from their legs, lungs, and body are (e.g. “How heavy is your breathing?”) [19,22].

The affective-motivational dimension was assessed with the Feeling Scale (FS) [23] and the Felt Arousal Scale (FAS) [24]. These instruments were used to investigate the individuals’ affective state (core affect) before the start of each trial (baseline) and in response to exercise (in-process). The FS is a single-item affect scale (11 points) varying from − 5 (very bad) to + 5 (very good). The FS has been developed to assess the affective valence/hedonic tone (i.e. degree of unpleasantness or pleasantness) of emotional responses during exercise. The FAS is a single-item arousal scale varying from 1 (low arousal) to 6 (high arousal) to investigate one's perceived activation during exercise. Both FS and FAS have been proven valid and reliable instruments to measure the affective responses to exercise (for details, see Ekkekakis et al. [25]). In addition to affective valence and arousal, dominance/control, the third dimension of affective responses was rated using a validated visual category scale with graphical anchors with “0” representing no control and “10” representing high control [26]. Boredom as well as motivation was assessed via two CR-10 scales with “0” representing “not at all bored/motivated” and 10 “very bored/motivated.”

Attentional focus was subsumed under the cognitive-evaluative responses. It was assessed with a one-dimensional scale measuring attention allocation with a score between 0 and 100. Associative thoughts were defined as those directed toward the motor task and the bodily symptoms associated with their completion (e.g. exercise-related symptoms like muscle pain or heaviness of breathing, discomfort, perceived effort). A value of “100” on this scale represents associative thoughts, only. Dissociative thoughts were defined as thoughts that are directed toward factors unrelated to the motor task of cycling (e.g. the environment, daydreaming, etc.) with a value of 0% representing only dissociative thoughts [27]. Furthermore, the cognitive-evaluative dimension included the assessment of the cognitive aspect of an action crisis, i.e. the decisional conflict between task disengagement and further goal pursuit (“I am in a serious conflict between proceeding to cycle or to quit”) ranging from 0 (“no agreement at all”) to 10 (“full agreement”) adapted from Brandstätter and Schüler [28].

### 2.5. Time perception

Two different prospective methods to assess the participants` time perception were applied. In a prospective time perception paradigm, participants know in advance that a time duration had to be judged later on, e.g. estimating the exercise duration after exercise cessation or producing a duration of 1 min after the occurrence of a stimulus. In a retrospective time perception paradigm, the person does not know in advance that a time duration has to be estimated later on. Retrospective paradigms are not feasible in cross-over studies, where time perception is repeatedly investigated.

#### 2.5.1. Time production

Following the ratings of perceived fatigue as well as the perceptual-discriminatory, affective-motivational, and cognitive-evaluative responses to exercise, participants were instructed to produce a time of exactly 1 min (time production paradigm) during the TTE cycling trials. For the time production paradigm, the absolute differences in seconds from the standard (1 min) can be compared within and between the experimental trials. The time production task can be viewed as an attention demanding cognitive dual task.

#### 2.5.2. Time estimation

Second, participants were asked to estimate the time duration of their TTE trial in minutes and seconds immediately after they stopped cycling at volitional exhaustion and the final ratings were completed (time estimation paradigm). The ratio of perceived time by elapsed time (time duration judgment ratio) was calculated to allow comparison of time perception between the individual TTE trials. In this regard, a time ratio > 1 means that time “crawls,” i.e. perceived time is longer than clock time because the “internal clock” runs faster. In contrast, a ratio < 1 means that time “flies,” i.e. perceived time is shorter than chronological time, because the “internal clock” runs slower [29]. Further, to compare time perception assessed with the two methods, the ratio of produced time duration by chronological time duration was also calculated. Both studies included the time production and the time estimation methods. The only difference between the studies was that participants in study 2 were only informed by the study material about the time estimation task after exercise cessation, and in study 1 participants were additionally instructed by the investigator before each TTE trial that a time duration estimation after exercise cessation has to be given.

### 2.6. Statistical analyses

After checking distribution for normality, paired t-tests were used to analyze the effect of caffeine on TTE as well as HR during exercise. To test the effect of caffeine on the dynamics of perceived fatigue as well as the perceptual-discriminatory, affective-motivational, and cognitive-evaluative responses to exercise, as well as time duration perception, repeated measures analyses of variance (RM-ANOVAs) using the within-subject factors condition (CAF vs. PLA) and time of measurement (time intervals), as well as their interaction (condition × time), were used. Therefore, ratings 1 min after the start (START) of the TTE trial, before exhaustion (PRE-EX, measurement time point defined by the last rating before task failure of the shortest trial), and at exhaustion (EX) were included in the analysis in the RM-ANOVAs. To evaluate whether perceived time differed from chronological time, separate RM-ANOVAs using the within-subject factors condition (CAF vs. PLA) and type of time duration (chronological vs. perceived time duration) as well as the condition × type were applied. In addition, for the time production paradigm, the interactions (type × time, type × condition × time) with the time points of measurement (START vs. PRE-EX) were investigated. Habitual caffeine consumption per kg body weight, assessed via a standardized questionnaire [16], was considered as covariate to control for a respective bias in the responses to caffeine [18]. If data violated the assumption of sphericity, Greenhouse-Geisser corrected p-values were reported. In case of significant main effects or interactions, post-hoc comparisons with Bonferroni corrections were performed. Partial eta squared *(η*_*p*_^*2*^, ≥ 0.01 = small effect, ≥ 0.06 = medium effect, ≥ 0.14 = strong effect) or Cohen’s *d* (≥ 0.2 = small effect, ≥ 0.5 = medium effect, ≥ 0.8 = strong effect) was used to determine the size of the effects [30].

## 3. Results

### 3.1. Study 1 (2.5 mg*kg^−1^)

Analysis of the nutrition protocols showed that average dietary caffeine intake before the experimental trials was identical for the caffeine and placebo condition (CAF: 63 ± 74 mg, PLA: 63 ± 74 mg). An important requirement of the study was that the participants should maintain their normal diet, including caffeinated beverages and food. Due to a number of participants habitually consuming caffeinated beverages or chocolate only in the afternoon or evening hours, 8 participants had a caffeine abstinence of ≥12 h before the measurement sessions took place, e.g. if they habitually consumed a coffee in the afternoon and the experimental testing was carried out in the morning hours.

#### 3.1.1. Performance and physiological data

No significant differences between the CAF and PLA trials was evident for TTE, absolute HR (statistical trend toward a higher HR for CAF), and relative HR (Table 3). The results did not change after removing the participant that reported no habitual dietary caffeine intake. Further, subsample analysis for the participants (*N* = 26) that habitually consumed dietary caffeine within 12 h prior the testing sessions showed a statistical trend for PLA being superior to CAF (1350 ± 954 s vs. 1145 ± 510, F_*(1,24)*_ = 4.051, *p* = 0.055, *η_p_*^*2*^ = 0.144). Post-hoc power analysis showed that this subsample analysis was well powered (1-β error probability: 0.999). On the contrary, subgroup analysis of the eight participants with caffeine abstinence of ≥12 h before the experimental trials were under-powered (1-β error probability: 0.069). In this subsample, CAF had also no significant effect on TTE (CAF vs. PLA: 1176 ± 682 s vs. 1076 ± 458 s, F_*(1,6)*_ = 0.015, *p* = 0.908, *η_p_^2^* = 0.002). Sample size calculation resulted in *N* = 486 participants that would be required to statistically prove the very small (irrelevant) effect of caffeine on TTE in low-to-moderate caffeine consumers that are “voluntary caffeine abstinent ≥12 h.”

**Table 3.** Means ± standard deviations and RM-ANCOVA statistics (covariate: habitual caffeine consumption) for the comparison of time to exhaustion (TTE), average absolute heart rate (HR), and relative HR (percentage of individual peak HR) for the caffeine (CAF) and placebo condition (PLA) in study 1 (2.5 mg*kg^−1^ caffeine). Due to technical reasons, HR was statistically evaluated for *N* = 33.

	CAF	PLA	ANCOVA statistics condition effect
TTE [s]	1154 ± 536	1279 ± 853	F*_(1,33)_* = 2.136, p = 0.153, *^η^_p_^2^= 0.061*
HR [min^−1^]	182 ± 10	180 ± 10	F*_(1,31) _*= 2.705, p = 0.110, *^η^_p_^2^= 0.080*
Relative HR [%]	93 ± 3	92 ± 4	F*_(1,31)_* = 2.521, p = 0.123, *^η^_p_^2^ = 0.075*

#### 3.1.2. Rating scales

A main effect of time was found for perceived fatigue, effort, physical strain, affective valence, arousal, dominance, motivation, boredom, and decisional conflict (Table 4). During the two trials, effort, physical strain, and arousal increased, while affective valence turned from positive to negative. Feelings of dominance/control, boredom, and motivation progressively declined, while the focus of attention became gradually associative and the decisional conflict increased. However, neither a significant main effect of condition nor a time × condition interaction was found for perceived fatigue as well as for the perceptual-discriminatory, affective-motivational, and cognitive-evaluative responses to exercise. The results did not change after removing the participant that reported no habitual caffeine consumption. The subsample analysis also yielded neither a main effect of condition nor a condition × time interaction effect, neither for the “abstinent” nor for the “non-abstinent” subgroup.

**Table 4.** Means ± standard deviations and RM-ANCOVA statistics (covariate: habitual caffeine consumption) for the different perceptual responses across the experimental trials of study 1 (2.5 mg*kg^−1^ caffeine). CAF: 2.5 mg*kg^−1^ caffeine, PLA: placebo, START: rating 1 min after start, PRE-EX: last rating during the trial, individual sampling time defined by the shortest individual trial, EX = rating at exhaustion.

Study 1 (2.5 mg*kg^−1^)		Time point			ANCOVA statistics		
	Condition	START	PRE-EX (iso-time)	EX	Effect of condition	Effect of time point	Effect of condition × time point
Perceived fatigue (0–10)	CAF	3.5 ± 1.8	8.3 ± 1.2	9.2 ± 1.0	F*_(1,33)_* = 1.095, p = 0.303, *η_p_^2^ = 0.032*	F*_(1.4,46.7)_* = 95.183, p < 0.001, *η_p_^2^ = 0.743*	F*_(2,66)_* = 0.381, p = 0.685, *η_p_^2^ = 0.011*
	PLA	3.3 ± 1.7	7.9 ± 1.4	9.1 ± 1.0			
Effort (6–20 BORG Scale)	CAF	11.3 ± 2.3	17.5 ± 2.0	19.5 ± 1.1	F*_(1,33)_* = 0.233, p = 0.632, *η_p_^2^ = 0.007*	F*_(1.6,52.6) _*= 100.468, p < 0.001, *η_p_^2^ = 0.753*	F*_(2,66)_* = 0.036, p = 0.965, *η^2^_p_* * = 0.001*
	PLA	11.4 ± 2.1	17.4 ± 2.0	19.3 ± 1.2			
Physical strain (6–20 BORG Scale)	CAF	10.3 ± 2.1	16.7 ± 2.0	18.7 ± 1.2	F*_(1,33)_* = 2.555, p = 0.120, *η_p_^2^ = 0.072*	F*_(1.7,54.8) _*= 122.539, p < 0.001, *η_p_^2^ = 0.788*	F*_(2,66) _*= 0517, p = 0.599, *η_p_^2^= 0.015*
	PLA	10.4 ± 2.1	16.5 ± 1.9	18.4 ± 1.0			
Affective valence (+5 – −5)	CAF	2.5 ± 1.2	−1.2 ± 2.1	−2.2 ± 2.1	F*_(1,33)_* = 0.517, p = 0.477, *η_p_^2^ = 0.015*	F*_(1.5,48.4)_* = 64.945, p < 0.001, *η_p_^2^_p_^2^ = 0.663*	F*_(2,66)_* = 1.337, p = 0.270, *η_p_^2^= 0.039*
	PLA	2.4 ± 1.3	−1.0 ± 2.1	−2.0 ± 2.3			
Arousal (1–6)	CAF	3.7 ± 1.1	4.8 ± 1.0	4.9 ± 1.2	F*_(1,33)_* = 1.953, p = 0.172, *η_p_^2^ = 0.056*	F*_(1.3,42.3)_* = 3.727, p = 0.029, *η_p_^2^ = 0.101*	F*_(2,66)_* = 0.390, p = 0.679, *η_p_^2^ = 0.012*
	PLA	3.9 ± 1.0	4.9 ± 1.2	4.9 ± 1.2			
Dominance/control (0–10)	CAF	7.2 ± 1.5	4.8 ± 2.1	3.7 ± 2.4	F*_(1,33)_* = 0.044, p = 0.835, *η_p_^2^ = 0.001*	F*_(1.5,47.9)_* = 130.438, p < 0.001, *η_p_^2^ = 0.561*	F*_(2,66)_* = 0.020, p = 0.980, *η_p_^2^= 0.001*
	PLA	7.1 ± 1.4	4.7 ± 2.1	3.6 ± 2.1			
Boredom (0–10)	CAF	1.5 ± 1.7	0.9 ± 1.6	0.9 ± 1.7	F*_(1,33)_* = 0.005, p = 0.941, *η_p_^2^ < 0.001*	F*_(1.5,51.0)_* = 3.505, p = 0.049, *η_p_^2^ = 0.096*	F*_(2,66)_* = 0.219 p = 0.804, *η_p_^2^ = 0.007*
	PLA	1.3 ± 1.7	1.2 ± 2.1	0.5 ± 1.0			
Motivation (0–10)	CAF	7.1 ± 1.8	4.4 ± 2.4	3.6 ± 2.6	F*_(1,33)_* = 0.322, p = 0.574, *η_p_^2^ = 0.010*	F*_(1.5,49.5)_* = 28.184, p < 0.001, *η_p_^2^ = 0.461*	F*_(2,66)_* = 1.206, p = 0.306, *η_p_^2^= 0.035*
	PLA	7.2 ± 1.3	4.7 ± 2.2	4.1 ± 2.7			
Attentional focus (0–100)	CAF	56.6 ± 21.7	80.0 ± 16.4	90.0 ± 13.3	F*_(1,33)_* = 0.327, p = 0.571, *η_p_^2^*η* = 0.010*	F*_(1.3,41.5)_* = 17.312, p < 0.001, *η_p_^2^ = 0.344*	F*_(1.6,53.0)_* = 1.359, p = 0.263, *η_p_^2^ = 0.040*
	PLA	56.9 ± 26.8	84.3 ± 14.2	94.0 ± 6.9			
Decisional conflict (0–10)	CAF	1.6 ± 1.5	7.2 ± 2.1	8.7 ± 1.4	F*_(1,33)_* = 0.657, p = 0.423, *η_p_^2^ = 0.020*	F*_(1.5,50.4) _*= 151.328, p < 0.001, *η_p_^2^ = 0.821*	F*_(1.6,53.8) _*= 1.152, p = 0.315, *η_p_^2^* *_p_^2^ = 0.034*
	PLA	1.4 ± 1.6	6.5 ± 2.5	8.8 ± 1.5			

#### 3.1.3. Time perception

##### 3.1.3.1. Time production

**Figure 2.**
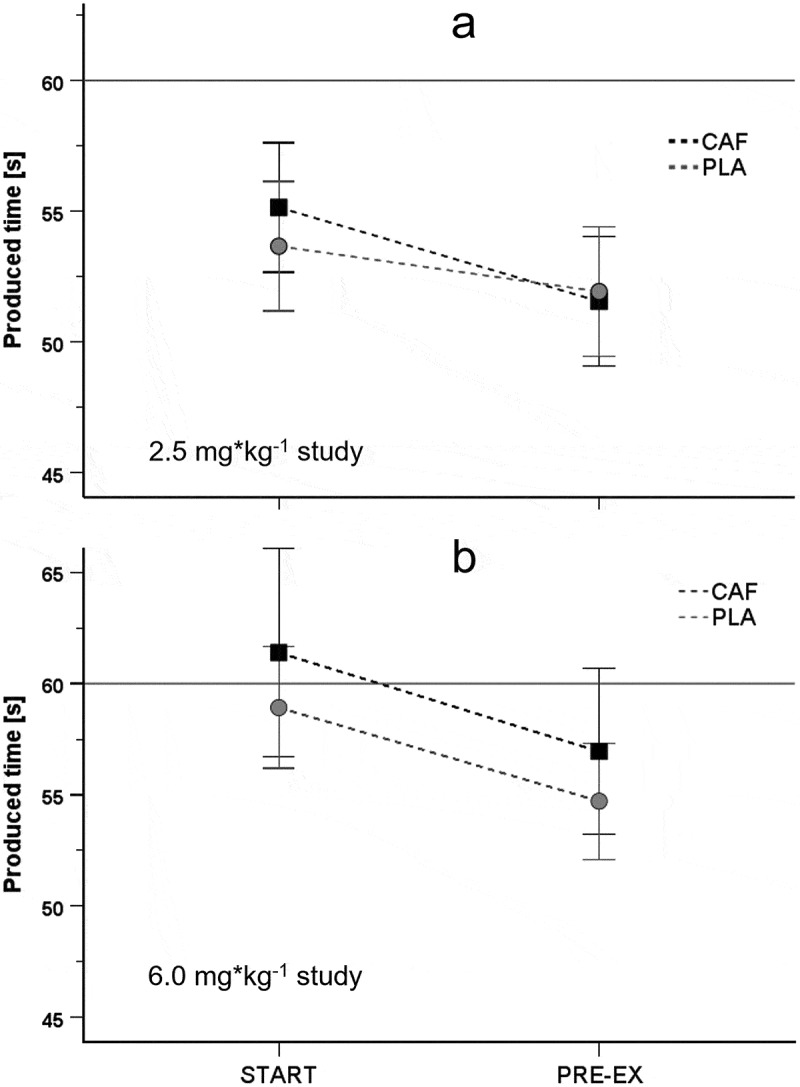
Time production at the beginning and the end of the TTE trials in study 1 (2.5 mg*kg^−1^ caffeine, panel A) and study 2 (6 mg*kg^−1^ caffeine, panel B). Black squares: caffeine condition, gray circles: placebo condition, solid gray line represents the standard (1 min) that had to be produced. START: time production 3 min after the start, PRE-EX: final time production before exhaustion.

Three participants stopped cycling before a second time production during exercise. Thus, only data of 32 participants were used to analyze the dynamics of time production in the CAF and PLA trial. No interaction of the experimental condition (CAF vs. PLA) with the difference between chronological and produced time was found (condition × type interaction: F*_(1,123)_ *= 2.014, *p* = 0.196, *η_p_^2^* = 0.002). Also, no condition (CAF vs. PLA) × type (chronological vs. produced) × time (START vs. PRE-EX) interaction was evident (F_*(1,123)*_ = 0.551, *p* = 0.459, all *η_p_^2^* = 0.004). However, irrespective of the supplement condition (CAF vs. PLA) as well as the time of measurement (START vs. PRE-EX), the effect of type (chronological vs. produced time) was significant. That means produced time was shorter than chronological time (average difference: 13.6 s, Bonferroni-corrected 95% CI: 11.4–16.5 s; F_*(1,123)*_ = 64.269, *p* < 0.001, *η_p_^2^* = 0.343, Figure 2). In addition, a significant type × time (START vs. PRE-EX) interaction was evident (F*_(1,123)_ *= 4.523, *p* = 0.035, *η_p_^2^* = 0.035) meaning that produced time significantly decreased during the trials by about 10% (average difference between START and PRE-EX: 5.3 s, 95% CI: 0.035–10.3 s, *p* = 0.035).

##### 3.1.3.2. Time estimation

Caffeine had no effect on the difference between chronological and estimated time duration (condition × type interaction: F_*(1,33)*_ = 0.859, *p* = 0.361, *η_p_^2^* = 0.025). Irrespective of the supplement condition, there was a trend (F*_(1,33)_ *= 2.337, *p* = 0.136, *η_p_^2^* = 0.066) for a difference between chronological and estimated time (average difference: 142.0 s, Bonferroni-corrected 95% CI: 11.5–272.4 s). Accordingly, the ratio of estimated by chronological time was 0.96 (adjusted 95% CI: 0.85–1.07) and 0.92 (adjusted 95% CI 0.79–1.04) for the CAF and PLA condition, respectively, pointing to an underestimation of the real exercise trial durations.

The results did not substantially change neither after removing the participant that reported no habitual caffeine consumption nor for the subsample analyses.

### 3.2. Study 2 (6 mg*kg^−1^)

Analysis of the nutrition protocols showed that average dietary caffeine intake before the experimental trials was similar for the caffeine and placebo condition (CAF: 22 ± 51 mg, PLA: 26 ± 53 mg, *p* = 0.407; *r* = 0.949, *p* < 0.001). An important requirement of the study was that the participants should maintain their normal diet. Due to a number of participants habitually consuming caffeinated beverages or chocolate only in the afternoon or evening hours, 12 participants had a caffeine abstinence of ≥12 h before the experimental measurements took place.

### 3.3. Performance and physiological data

No differences between the CAF and PLA trials were evident for TTE, absolute HR, and relative HR (Table 5). The results did not change after removing the participant that reported no habitual dietary caffeine intake. Further, subsample analysis for the participants (*N* = 8) that consumed dietary caffeine within 12 h prior the testing sessions showed no significant effect of CAF on TTE (2589 ± 1350 s vs. 2480 ± 1803, F_*(1,6)*_ = 0,005 *p* = 0.944, *η_p_^2^*  = 0.001). Post-hoc power analysis showed that the subsample analysis was underpowered (1-β error probability: 0.115) and sample size calculation resulted in *N* = 145 participants that would be required to statistically prove the very small (irrelevant) effect of caffeine on TTE in this subsample. On the contrary, subgroup analysis of the 12 participants with caffeine abstinence of ≥12 h before the experimental trials was well-powered (1-β error probability: 0.906). In this subsample, CAF had also no significant effect on TTE (CAF vs. PLA: 1523 ± 998 s vs. 1320 ± 763 s, F_*(1,10)*_ = 0.885, *p* = 0.369, *η_p_^2^*  = 0.081).

**Table 5.** Means ± standard deviations and RM-ANCOVA statistics (covariate: habitual caffeine consumption) for the comparison of time to exhaustion (TTE), average absolute (HR), and relative HR (percentage of individual peak HR) for the caffeine (CAF) and placebo condition (PLA) in study 2 (6 mg*kg^−1^). Due to technical reasons, HR was evaluated for *N* = 20.

	CAF	PLA	ANCOVA statistics condition effect
TTE [s]	1915 ± 1218	1754 ± 1341	F*_(1,19)_* = 0.773, p = 0.390, *η_p_^2^ = 0.039*
HR [min^−1^]	173 ± 14	174 ± 12	F*_(1,18)_* < 0.001, p = 0.993, *η_p_^2^ < 0.001*
relative HR [%]	91 ± 5	91 ± 3	F*_(1,18)_* = 0.001, p = 0.982, *η_p_^2^* *< 0.001*

### 3.4. Rating scales

Two participants stopped cycling before a second rating during the exercise, thus only data of 19 participants were used to analyze the rating scale data.

A main effect of time was found for perceived fatigue, effort, physical strain, affective valence, arousal, dominance, motivation, boredom, attentional focus, and decisional conflict (Table 6). During the two trials, effort, physical strain, and arousal increased, while affective valence turned from positive to negative. Feelings of dominance/control, boredom, and motivation progressively declined, while the focus of attention became gradually associative and the decisional conflict increased. However, neither a significant main effect of condition nor a time × condition interaction was found for all perceptual responses. The results did not change after removing the participant that reported no habitual caffeine consumption. As mentioned, although the participants were not restricted with regard to their caffeine consumption, 12 participants had a caffeine abstinence of ≥12 h before the measurement sessions took place. A subsample analysis yielded neither a significant main effect of condition nor a condition × time interaction effect for the subgroups with one exception. For the feeling of control, a small significant condition × time interaction effect was evident. Participants that were “restricted” for ≥12 h showed a slightly stronger decline during cycling in the CAF condition compared to PLA.

**Table 6.** Means ± standard deviations and RM-ANCOVA statistics (covariate: habitual caffeine consumption) for the different perceptual responses across the experimental trials of study 2 (6 mg*kg^−1^) (because three participants were not able to sustain more than 5 min data of *N* = 19 were analyzed). CAF: 6 mg*kg^−1^ caffeine, PLA: placebo, START: rating 1 min after start, PRE-EX: last rating during the trial, individual sampling time defined by the shortest individual trial, EX = rating at exhaustion.

Study 2 (6 mg*kg^−1^)		Time point			ANCOVA statistics		
	Condition	START	PRE-EX (iso-time)	EX	Effect of condition	Effect of time point	Effect of condition × time point
Perceived fatigue (0–10)	CAF	2.7 ± 1.9	7.6 ± 1.8	9.3 ± 0.9	F*_(1,33)_* = 0.041, p = 0.843, *η_p_^2^= 0.002*	F*_(1.2,19.8)_* = 56.544, p < 0.001, *η_p_^2^ = 0.769*	F*_(2,34)_* = 0.649, p = 0.529, *η_p_^2^= 0.037*
	PLA	3.1 ± 2.1	7.9 ± 2.3	9.0 ± 1.7			
Effort (6–20 BORG Scale)	CAF	11.1 ± 1.7	18.0 ± 1.8	19.4 ± 2.7	F*_(1,17)_* = 0.035, p = 0.853, *η_p_^2^= 0.002*	F*_(2,34)_* = 38.559, p < 0.001, *η_p_^2^ = 0.694*	F*_(2,34)_* = 1.729, p = 0.193, *η_p_^2^ = 0.092*
	PLA	11.1 ± 2.4	18.3 ± 1.7	19.2 ± 2.0			
Strain (6–20 BORG Scale)	CAF	10.1 ± 2.0	17.3 ± 2.2	18.7 ± 2.2	F*_(1,17) _*= 0.093, p = 0.764, *η_p_^2^ = 0.005*	F*_(2,34)_* = 43.440, p < 0.001, *η_p_^2^ = 0.719*	F*_(2,34) _*= 0.584, p = 0.563, *η_p_^2^ = 0.033*
	PLA	10.5 ± 2.1	17.6 ± 2.0	18.5 ± 1.8			
Affective valence (+5 – −5)	CAF	3.1 ± 1.3	−0.6 ± 2.4	−1.5 ± 2.9	F_*(1,17)*_ = 0.122, *p* = 0.731, *η_p_^2^* = 0.007	F_*(1.5,25.4)*_ = 28.814, *p* < 0.001, *η_p_^2^* = 0.629	F*_(1.5,24.8)_* = 1.358, *p* = 0.271, *η_p_^2^* = 0.074
	PLA	2.5 ± 1.9	−0.7 ± 2.9	−1.3 ± 2.6			
Arousal (1–6)	CAF	3.7 ± 1.2	5.3 ± 1.2	5.1 ± 1.7	F*_(1,17)_* = 0.371, *p* = 0.551, *η_p_^2^* = 0.021	F*_(1.1,19.2)_* = 29.247, *p* < 0.001, *η_p_^2^* = 0.632	F*_(1.2,20.2)_* = 1.253, *p* = 0.298, *η_p_^2^* = 0.069
	PLA	3.9 ± 1.1	5.4 ± 1.3	5.5 ± 1.4			
Dominance/control (0–10)	CAF	8.1 ± 2.0	5.2 ± 2.8	4.7 ± 3.7	F_*(1,17*_) = 0.510, *p* = 0.485, *η_p_^2^* = 0.029	F(1.4,23.3) = 15.061, *p* < 0.001, *η_p_^2^* = 0.470	F(1.9,25.3) = 1.261, *p* = 0.296, *η_p_^2^* = 0.069
	PLA	8.0 ± 1.7	5.3 ± 2.8	4.6 ± 3.3			
Boredom (0–10)	CAF	1.8 ± 2.3	1.6 ± 2.5	1.8 ± 2.1	F*_(1,17)_* = 0.254, *p* = 0.621, *η_p_^2^* < 0.015	F*_(2,17)_* = 25.502, *p* = 0.002, *η_p_^2^*η = 0.301	F*_(1.3,22.3)_* = 0.056 *p* = 0.946, *η_p_^2^* = 0.003
	PLA	1.8 ± 0.7	0.7 ± 1.8	0.6 ± 1.6			
Motivation (0–10)	CAF	8.2 ± 1.5	5.3 ± 2.9	3.6 ± 3.5	F*_(1,17)_* = 0.450, *p* = 0.51, *η_p_^2^* = 0.026	F*_(1.4,24.4) _*= 18.146, *p* < 0.001, *η_p_^2^* = 0.516	F*_(2,34)_* = 0.268, *p* = 0.767, *η_p_^2^* = 0.016
	PLA	7.6 ± 1.8	4.5 ± 3.5	3.3 ± 3.2			
Attentional focus (0–100)	CAF	52.1 ± 27.6	73.7 ± 26.3	81.6 ± 27.7	F_*(1,17)*_ = 0.493, *p* = 0.492, *η_p_^2^* = 0.028	F*_(1.3,22.2)_* = 4.63, *p* = 0.034, ηp [2] = 0.214	F*_(1.4,25.0)_* = 0.296, *p* = 0.679, *η_p_^2^* = 0.017
	PLA	56.3 ± 25.4	71.1 ± 31.8	75.8 ± 34.9			
Decisional conflict (0–10)	CAF	1.2 ± 1.3	6.4 ± 2.2	8.8 ± 2.0	F*_(1,17)_* = 1.043, p = 0.321, *η_p_^2^ = 0.058*	F*_(2,34)_* = 35.380, p < 0.001, *η_p_^2^ = 0.675*	F_(*2,34) *_*= 0.992, p = 0.381, η_p_^2^ = 0.055*
	PLA	1.2 ± 1.9	6.8 ± 2.8	7.8 ± 2.9			

### 3.5. Time perception

#### 3.5.1. Time production

Two participants stopped cycling before a second time production within the exercise. Thus, only data of 19 participants were used to analyze the dynamics of time production precision in the CAF and PLA trial. No interaction of experimental condition (CAF vs. PLA) with the difference between chronological and produced time was found (condition × type interaction: F_*(1,71)*_ = 2.014, *p* = 0.160, *η_p_^2^* = 0.028). Also, no condition × type × time (START vs. PRE-EX) was evident (F_*(1,71)*_ = 0.005, *p* = 0.943, *η_p_^2^* < 0.001). However, irrespective of the supplement condition (CAF vs. PLA) as well as the time of measurement (START vs. PRE-EX), the effect of type (chronological vs. produced time) was significant, meaning that produced time was shorter than chronological time (average difference: −4.0 s, Bonferroni-corrected 95%: −7.3–0.7 s; F*_(1,71)_ *= 9.652, *p* = 0.003, *η_p_^2^* = 0.120, Figure 2). In addition, a significant type × time interaction was evident (F_*(1,71)*_ = 6.804, *p* = 0.011, *η_p_^2^* = 0.087). Post-hoc analysis revealed that, while there was no difference between chronological and produced time at the beginning (average difference at START: −0.3 s; Bonferroni-corrected 95% CI: −5.0–4. 4 s; *p* = 0.893), produced time shortened at the end of the trials (average difference at PRE-EX: −8.3 s; Bonferroni-corrected 95% CI: −13.0–3.7 s; *p* < 0.001).

#### 3.5.2. Time estimation

Caffeine had no effect on the difference between chronological and estimated time duration (condition × type interaction: F_*(1,19)*_ = 0.158, *p* = 0.696, *η_p_^2^* = 0.008). Irrespective of the supplement condition, estimated time was significantly shorter than chronological TTE (F_*(1,19)*_ = 12.190, *p* = 0.002, *η_p_^2^* = 0.391, average difference: 398.0 s, Bonferroni-corrected 95% CI: 223.0–573.0 s). Accordingly, the ratio of estimated by chronological time was 0.73 (adjusted 95% CI: 0.60–0.86) and 0.75 (adjusted 95% CI 0.61–0.89) for the CAF and PLA condition, respectively, proving the subjective underestimation of the real exercise trial duration in both conditions.

The results did not change neither after removing the participant that reported no habitual caffeine consumption nor for the subsample analyses.

## 4. Discussion

The results of both studies indicate that, without a prior caffeine restriction phase, TTE, perceived fatigue, as well as the perceptual-discriminatory, affective-motivational, and cognitive-evaluative responses to exercise were not significantly affected by caffeine ingestion during constant-load cycling until exhaustion in active subjects. Moreover, no effect of caffeine intake on time perception was found. Additional statistical analyses using sex as a covariate or stratifying the data for sex resulted in no divergent findings.

While most studies investigating the effects of caffeine on endurance performance employed a prior phase of caffeine restriction and thereby might have induced a kind of withdrawal symptomology, participants of the present study were intentionally asked not to deviate from their normal diet and to proceed with habitual caffeine consumption. It has been shown that a restriction phase in regular caffeine users can produce side-effects including symptoms of fatigue, mood disturbances, and decrements in cognitive performance (e.g. Addicott and Laurienti [31]). A review on this topic provides further support for the so-called withdrawal reversal hypothesis for cognitive task performance and mood. Accordingly, the net beneficial psychostimulant effects of caffeine are almost wholly attributable to the reversal of adverse withdrawal effects associated with short periods of abstinence in regular caffeine users [12]. Interestingly, these findings have only rarely been systematically investigated and considered as an explanation for the potentially performance-enhancing effects of caffeine during endurance and motor tasks in general. Given that the ergogenic effect of caffeine on endurance performance is thought to be mainly attributable to its pharmacological actions in the central nervous system, it might be possible that the effects of caffeine on endurance performance can be explained by the same mechanism. Since both studies presented in this article have not found an effect of caffeine on the dependent variables, it is cautiously concluded that the ergogenic effects of caffeine on constant-load cycling endurance performance might be modulated by the reversal of withdrawal symptoms after short periods of caffeine restriction.

Moreover, no effect of caffeine on time production has been found, since there were no differences in time production precision between the PLA and CAF conditions in both studies. Although, to the authors’ knowledge, the effect of caffeine on time production during exercise was not investigated to date, there are a few studies investigating the effects of caffeine on cognitive performance during or after a fatiguing motor task. Equivocal findings regarding reaction times were found for caffeine supplementation, and again, potential withdrawal reversal effects may have biased positive findings [32–35]. While caffeine had no effect on time production, progressive fatigue seemed to worsen time perception in all trials, since produced time became increasingly shorter during the cycling TTE trials. This is in line with findings indicating that (maximal) physical exertion making time “crawl,” i.e. elapsed time is overestimated [36–40]. The scalar expectancy theory of time perception explains how psycho-physiological processes can modulate time perception. Fatigue and associated arousal levels can lead to an increase in the pacemaker rate of the internal clock [41]. Further, attentional shifts to time perception, facilitated by non-goal oriented activity or aversive states like boredom [42], can result in an earlier/faster accumulation of these pacemaker pulses [41]. Both an increase in pacemaker rate and attentional shifts to time perception during the exercise can have resulted in time distortion in our studies. Since boredom ratings were low and even decreased during the cycling, boredom does not seem to play a role in time perception during cycling until exhaustion. Instead, the significant increase in negative affect and arousal as well as an attentional shift induced by exercise-related symptoms like muscle pain or heaviness of breathing, discomfort, and perceived effort may explain the shorter time productions at the end of the exercise in our studies.

In contrast to the results from the time production paradigm, estimated time duration after the trials was shorter than chronological time in both conditions. This finding speaks for a subjectively faster passing of time, which is in contrast to the perception assessed with the time production paradigm during the trials and most findings in the literature. Here, it should be noted that time duration estimation and time production are methodologically different paradigms and draw on partially different cognitive resources [43]. Although both methods used in our studies are considered as prospective time perception methods, i.e. the participants knew in advance that a time duration judgment has to be made after exercise cessation, the time duration estimations after the trials may exhibit some features of retrospective duration judgments. For these retrospective time duration judgments, memory for events and contextual changes play a major role, unless a person has little information to process, which may frequently induce feelings of boredom. In these cases, also attention may affect retrospective time estimation [44]. In a meta-analytic review, Block and Zakay [44] have shown that prospective duration judgments are longer, when compared to retrospective judgments. The prospective time duration judgment ratios (i.e. estimated duration by chronological duration) retrieved from the meta-analysis for moderate and long durations were similar to the judgment ratios in the 2.5 mg*kg^−1^ caffeine study experiment (0.96 and 0.92 for the CAF and PLA condition, respectively), while the ratios in our 6 mg*kg^−1^ caffeine study were smaller (average difference for CAF: 0.23, 95% CI 0.06–0.39, *p* = 0.005, Cohen’s *d*: 0.760; average difference for PLA: 0.17, 95% CI: −0.01–0.34, *p* = 0.043, Cohen’s *d*: 0.524) and corresponded to typical retrospective paradigm studies. These findings are most probably related to different investigator instructions. In the first study (2.5 mg*kg^−1^), participants were informed by the study material as well as orally by the investigator before each start that an estimation of the completed time to has to be carried out after the cycling, while they were only informed by the study material in the second study (6 mg*kg^−1^). While not being the primary aim of this investigation, results generally support the notion that attentional shifts affect both time production and time estimation judgments during and after endurance exercise.

### 4.1. Limitations

There is always a trade-off when using TTE but also time-trial paradigms. While TTE experiments lack in way ecological validity, they allow statistical comparisons at identical workload and time points during the experiments to elucidate the dynamics of perceived fatigue. Such comparisons are not possible with time-trial experiments.

Individual genotype (CYP1A2 and ADORA2A polymorphisms) may have affected the metabolizing of caffeine and thus its ergogenic effect [45]. However, when excluding studies reporting a conflict of interest from the review of Barreto et al. [45], no effect of genotype was evident, which is in line with experimental findings that show no such variability in the ergogenic response to caffeine [46].

Menstrual cycle phase may potentially have biased the results, but statistical control of self-reported menstrual cycle phase did not change the results. The lack of determination of female sex hormones must be stated as a limitation in this context, but a current study suggests that there is no statistically verifiable effect of caffeine on endurance performance or exercise-associated perception depending on the menstrual cycle phase [47]. Generally, current reviews indicate that neither TTE at sub-maximal exercise intensities [48] nor RPE at continuous aerobic exercise [49] is modulated over the menstrual cycle.

Although the participants were not restricted with regard to their caffeine consumption, some of them had their last dietary caffeine intake ≥12 h prior to the testing sessions, e.g. if they habitually consumed a coffee in the afternoon and the experimental testing was carried out in the morning hours. We therefore undertook subgroup analyses, which supported the assumptions from the total sample analyses, although it must be acknowledged that not all of statistical subsample tests were sufficiently powered. It is further worth noting that our participants were low to moderate habitual caffeine consumers, meaning that, even after an overnight “caffeine fast”, no pronounced pharmacological withdrawal effect should occur. They are further taking caffeine in different forms during different times of the day, e.g. some in the morning and others in the afternoon. This habitual consumption time in combination with the timing of the measurement sessions led, in some cases, to the abstinence ≥12 h. Nevertheless, these participants were not restricted in their daily “caffeine routine” and therefore no psychological withdrawal effect can be expected. Guaranteeing habitual caffeine intake before the measurement session would have required to manipulate the daily “caffeine routine” of the subjects or an exclusion criterion for those participants who, for instance, had time for the measurement in the morning and habitually consume caffeine in the afternoon. This would also have resulted in lower validity of results. In this respect it has to be considered that also exercise sessions and competitions take place in morning hours.

While we have critically argued that an overnight fast might have biased some results in the existing literature (which might be the case especially in heavy consumers), our aim was not explicitly to test individuals without caffeine restriction ≥12 h but to apply an ecological valid design, where they followed their normal “ad libitum” diet and felt no “psychological” withdrawal.

To address concerns that might be raised in this respect, we carried out a subsample analysis. The sub-sample analysis yielded no divergent results for TTE (as well as perceived fatigue). Further, caffeine should have stronger effects in caffeine-withdrawn subjects. The inclusion of such subjects in a study sample should assumingly also favor the statistical occurrence of a positive effect of caffeine. The conclusions from our study are therefore valid, even if participants who “voluntarily abstained” from caffeine were not excluded.

## 5. Conclusion

Data of the present experiments indicate that both dosages of caffeine had no effect on constant-load cycling endurance performance, perceived fatigue, as well as the perceptual-discriminatory, affective-motivational, and cognitive-evaluative responses to exercise in male and female participants who underwent no prior phase of caffeine restriction. Moreover, time perception was also not modulated by both doses of caffeine. Therefore, it is cautiously concluded that caffeine´s ergogenic effects on endurance performance might be partly explained by the reversal of adverse effects after short periods of caffeine restriction, typically applied prior to experiments dealing with this topic.
